# Supplementary material for: Polar cod in jeopardy under the retreating Arctic sea ice
Source: Commun Biol. 2019 Nov 7;2:407. doi: 10.1038/s42003-019-0649-2 (PMC6838109; doi:10.1038/s42003-019-0649-2)
Supplement: Supplementary file 1 — Reporting Summary [file 42003_2019_649_MOESM1_ESM.pdf]

nature research

Corresponding author(s): Mats Huserbråten

Last updated by author(s): Aug 30, 2019

Reporting Summary

Nature Research wishes to improve the reproducibility of the work that we publish. This form provides structure for consistency and transparency in reporting. For further information on Nature Research policies, see Authors & Referees and the Editorial Policy Checklist.

Statistics

For all statistical analyses, confirm that the following items are present in the figure legend, table legend, main text, or Methods section.

n/a	Confirmed	
☐	☒	The exact sample size (*n*) for each experimental group/condition, given as a discrete number and unit of measurement
☐	☒	A statement on whether measurements were taken from distinct samples or whether the same sample was measured repeatedly
☐	☒	The statistical test(s) used AND whether they are one- or two-sided<br>*Only common tests should be described solely by name; describe more complex techniques in the Methods section.*
☐	☒	A description of all covariates tested
☐	☒	A description of any assumptions or corrections, such as tests of normality and adjustment for multiple comparisons
☐	☒	A full description of the statistical parameters including central tendency (e.g. means) or other basic estimates (e.g. regression coefficient) AND variation (e.g. standard deviation) or associated estimates of uncertainty (e.g. confidence intervals)
☐	☒	For null hypothesis testing, the test statistic (e.g. *F*, *t*, *r*) with confidence intervals, effect sizes, degrees of freedom and *P* value noted<br>*Give P values as exact values whenever suitable.*
☒	☐	For Bayesian analysis, information on the choice of priors and Markov chain Monte Carlo settings
☒	☐	For hierarchical and complex designs, identification of the appropriate level for tests and full reporting of outcomes
☐	☒	Estimates of effect sizes (e.g. Cohen's *d*, Pearson's *r*), indicating how they were calculated

Our web collection on statistics for biologists contains articles on many of the points above.

Software and code

Policy information about availability of computer code

Data collection	All custom code used to collect data is referred to in reference list
Data analysis	All custom code used to analyze data is referred to in reference list

For manuscripts utilizing custom algorithms or software that are central to the research but not yet described in published literature, software must be made available to editors/reviewers. We strongly encourage code deposition in a community repository (e.g. GitHub). See the Nature Research guidelines for submitting code & software for further information.

Data

Policy information about availability of data

All manuscripts must include a data availability statement. This statement should provide the following information, where applicable:

- Accession codes, unique identifiers, or web links for publicly available datasets
- A list of figures that have associated raw data
- A description of any restrictions on data availability

Data analyzed in this study will be made available on request to the authors.

Field-specific reporting

Please select the one below that is the best fit for your research. If you are not sure, read the appropriate sections before making your selection.

☐ Life sciences ☐ Behavioural & social sciences ☒ Ecological, evolutionary & environmental sciences

For a reference copy of the document with all sections, see nature.com/documents/nr-reporting-summary-flat.pdf

Ecological, evolutionary & environmental sciences study design

All studies must disclose on these points even when the disclosure is negative.

Study description	Simulations were replicated over 28 spawning seasons of polar cod
Research sample	Pelagic trawl and echo-sounder surveys was used to sample the distribution and abundance of wild polar cod (Boreogadus saida)
Sampling strategy	Sampling was performed in regular grids by pelagic trawls and echo-sounder transects
Data collection	Data was collected on board research vessels by qualified technicians
Timing and spatial scale	Survey was performed between late August and late October every year between 1990-2017
Data exclusions	No data was excluded
Reproducibility	N/A
Randomization	N/A
Blinding	N/A

Did the study involve field work? ☒ Yes ☐ No

Field work, collection and transport

Field conditions	Typical weather in the Arctic Ocean
Location	Barents Sea
Access and import/export	Surveys were performed in a regular, pre-determined grid unless ice prevented the movement of the research vessel
Disturbance	Disturbance by ice was minimized by sampling at time of minimum ice cover

Reporting for specific materials, systems and methods

We require information from authors about some types of materials, experimental systems and methods used in many studies. Here, indicate whether each material, system or method listed is relevant to your study. If you are not sure if a list item applies to your research, read the appropriate section before selecting a response.

Materials & experimental systems

n/a	Involved in the study	
☒	☐	Antibodies
☒	☐	Eukaryotic cell lines
☒	☐	Palaeontology
☐	☒	Animals and other organisms
☒	☐	Human research participants
☒	☐	Clinical data

Methods

n/a	Involved in the study	
☒	☐	ChIP-seq
☒	☐	Flow cytometry
☒	☐	MRI-based neuroimaging

Animals and other organisms

Policy information about studies involving animals; ARRIVE guidelines recommended for reporting animal research

Laboratory animals	N/A
Wild animals	Wild polar cod were caught for the data analyzed in this study, and those caught in the pelagic trawls were killed according to common practice.
Field-collected samples	N/A
Ethics oversight	Data analyzed in this study was taken from a data-base not collected specifically for this study. However, the national organization responsible has approved the survey, performed as a ecosystem survey run annually ordered by the Norwegian directory for fisheries

Note that full information on the approval of the study protocol must also be provided in the manuscript.
